# Contemporary Profile and In-Hospital Outcomes of Decompensated Heart Failure in a Semi-Rural Setting in Cameroon: The Buea Heart Study

**DOI:** 10.5334/gh.1442

**Published:** 2025-06-23

**Authors:** Clovis Nkoke, Jean Jacques Noubiap, Siddikatou Djibrilla, Ali Abas, Ahmadou Musa Jingi, Ulrich Flore Nyaga, Gijo Thomas, Alain Menanga, Samuel Kingue, Anastase Dzudie

**Affiliations:** 1 University of Buea, Faculty of Health Sciences, Buea, Cameroon; 2 Division of Cardiology, Department of Medicine, University of California-San Francisco, San Francisco, California, United States; 3 Faculty of Medicine and Biomedical Sciences, University of Ngaoundere, Ngaoundere, Cameroon; 4 University of Bamenda, Faculty of Health Sciences, Bamenda, Cameroon; 5 Health Data Acumen, Nairobi, Kenya; 6 Department of Medicine, Edea Regional Hospital, Edea, Cameroon; 7 Avance Clinical Pty Ltd, Adelaide, Australia; 8 University of Yaounde, Faculty of Medicine and Biomedical Sciences, Yaounde, Cameroon; 9 Douala General Hospital, Douala, Cameroon

**Keywords:** Heart failure, semi-rural, mortality, length of stay, Cameroon, Africa

## Abstract

**Background:**

Available evidence suggests that the epidemiology of heart failure (HF) in sub-Saharan Africa (SSA) might be changing. However, there is a scarcity of contemporary data on the epidemiology and prognosis of hospitalized HF patients in Cameroon despite improvements in the treatment of HF and the changing epidemiology of HF in SSA in the last decade.

**Objective:**

To examine the contemporary characteristics, the in-hospital outcomes, and their predictors in patients hospitalized for decompensated HF in Buea, South West region of Cameroon.

**Methods:**

We conducted an observational prospective cohort study. We included consecutive patients hospitalized for HF from March 2021 to March 2024. Multivariate logistic regression analyses were performed to determine factors associated with in-hospital mortality and prolonged length of hospital stay (LOS). A p-value < 0.05 was considered statistically significant.

**Results:**

Out of the 477 patients included, 254 (53.2%) were females. The mean age was 60.3 ± 16.5 years. The most common co-morbidities were hypertension (55.6%), atrial fibrillation (20.8%), diabetes mellitus (17.6%), and chronic kidney disease (14.1%). The most common causes of heart failure were hypertensive heart disease (41.7%), ischemic heart disease (15%), cor pulmonale (11.9%), and dilated cardiomyopathy (9%). The median length of stay (LOS) was seven days. Factors that increased odds of prolonged LOS were atrial fibrillation (OR = 2.04, CI: 1.26–3.35; p = 0.005). Factors that reduced odds of prolonged LOS were valvular heart disease (VHD) (OR = 0.49, CI: 0.26–0.91; p = 0.023), systolic blood pressure (SBP) (OR = 0.99 per 1 mmHg increment, CI: 0.98–0.99; p = 0.005), and natremia (OR = 0.96 per 1 unit increment, CI: 0.93–0.99; p = 0.010). In-hospital mortality was 11.9%. Factors that increased odds of in-hospital mortality were VHD (OR = 2.40, CI: 1.02–5.64; p = 0.045) and dobutamine administration (OR = 4.37, CI: 1.11–17.16; p = 0.034). Factors that reduced odds of mortality were SBP (OR = 0.99, CI = 0.98–0.99; p = 0.033), natremia (OR = 0.93, CI: 0.89–0.97; p < 0.001), and glomerular filtration rate (OR = 0.98 per 1 unit increment, CI: 0.97–0.99; p = 0.001).

**Conclusion:**

The causes of HF are still predominantly hypertensive, but there is an increasing contribution of ischemic heart disease. There is a need to improve hypertension control and other risk factors for ischemic heart disease in SSA.

## Background

Heart failure (HF) is a major public health problem worldwide, including sub-Saharan Africa (SSA) (1). HF is associated with significant morbidity and mortality, and the burden is increasing in SSA due to the increasing urbanization and the rapid epidemiological transition with increasing risk factors for cardiovascular diseases and HF (2,3).

The THESUS-HF registry was the first registry to characterize causes, treatment, and outcome of heart failure in 1006 African patients, which was published more than a decade ago (4). The mean age of patients in the THESUS-HF was 52 years, and the in-hospital mortality was 4.2%. The most common causes of HF were hypertensive heart disease and rheumatic heart disease (RHD), accounting for 45.4% and 14.3% of all causes of AHF, respectively (4). Available evidence indicates that the epidemiology of HF in Africa is changing. In comparison to data prior to 2005, the THESUS-HF highlighted the following trends in the etiology of HF: a rise in ischemic heart disease (from 2% to 8%), a decrease in the recognition of rheumatic heart disease (from 22% to 17%), an increase in the importance of cardiomyopathies (from 20% to 29%), and a rise in hypertension (from 23% to 43%) (4,5). There also exists regional and geographic variation in HF. Heart failure patients in SSA are the youngest compared with patients in high-income countries (6,7). Reports from studies performed before 2005 in eight SSA countries demonstrated that up to 75% of cases of heart failure were non-ischemic in etiology (5). Ischemic heart disease, which is the predominant cause of HF in high-income countries and previously considered rare in SSA, is on the rise in SSA in line with the epidemiological transition in Africa, with a rise in the risk factors, such as hypertension, diabetes, and obesity (5,6). Recent studies that have specifically looked for evidence of ischemic heart disease have shown a higher prevalence than previously reported. A case-control study from Kenya recently indicated that ischemic heart disease was the second most common cause of HF (8). Similarly, another study from Kenya indicated that the prevalence of ischemic heart disease in African patients with HF with reduced ejection fraction (HFrEF) was 52.3% (9).

Hospitalization for HF is associated with increased risk of mortality (10). The mortality due to heart failure is highest in low-income countries, including SSA, compared to high-income countries. In the INTER-CHF, overall mortality was 16.5%: highest in Africa (34%) and India (23%), intermediate in Southeast Asia (15%), and lowest in China (7%), South America (9%), and the Middle East (9%) (7). Similarly, in the G-GHF, low-income countries recorded the highest age-standardized mortality (6). In the THESUS-HF registry, in-hospital mortality of HF was 4.2% (4). There have been significant recent advances in the pharmacological treatment of heart failure in the past decade, which have revolutionized HF treatment and improved the survival of patients with heart failure (11). But in Africa, affordability and availability are limitations to having access to these essential cardiac medications (12). Despite advancements in the pharmacological treatment and the changing epidemiology of HF in SSA, there are limited contemporary data on the characteristics, treatment, and outcome of patients hospitalized for acute decompensated HF in SSA. Moreover, most of the available data on HF in Cameroon comes from large urban cities. Data on HF in semi-rural areas can provide insights into how HF differs from urban centers. Semi-rural areas often have limited access to advanced diagnostic tools and specialized care. Also, the etiologies of heart failure vary significantly across different regions within Africa between semi-rural and urban settings.

The aim of this study was to describe the contemporary characteristics and the in-hospital outcome and its predictors in patients hospitalized for decompensated HF in a semi-rural setting in Cameroon. Data on heart failure in semi-rural African settings will help address specific regional health challenges and contribute valuable data for improving HF care across diverse populations within the continent.

## Methods

### Study design

This was an observational prospective cohort study conducted at the Buea Regional Hospital, Southwest Region of Cameroon, between March 2021 and March 2024.

### Setting

The Buea Regional Hospital is a 400-bed capacity secondary-level hospital that serves as a referral center in the Southwest Region of the country. It serves a population of about 300,000 inhabitants in the municipality and receives referred cases from other parts of the region. The most common socioeconomic activities in the region are farming and trading. The internal medicine unit is staffed with a neurologist and cardiologist and offers cardiovascular investigations such as electrocardiogram (ECG), Holter ECG, transthoracic echocardiogram, and conventional x-ray.

### Sampling

Consecutive patients hospitalized for decompensated heart failure between March 2021 and March 2024 were included in the study. We excluded patients who did not have echocardiography.

### Study population

We included all patients aged ≥18 years hospitalized during the study period who consented to take part in the study. Heart failure was diagnosed according to standard guidelines.

### Data collection

For each patient, we collected data on sociodemographic characteristics, clinical features (including physical examination findings and comorbidities), diagnosis, laboratory findings, electrocardiogram, echocardiogram, and hospital stay. The treatment outcome was assessed at the time of discharge from the hospital.

### Definition of terms

Prolonged length of stay: defined as a hospital stay of more than seven days.

In-hospital mortality: death of a patient with acute HF due to any cause, occurring after admission to the medical unit.

### Ethical Clearance

Ethics approval was obtained from the institutional review board of the Buea Regional Hospital acting as the local ethics committee (ref: 175/MPH/SWRDPH/BRH/IRB). Informed consent was obtained from patients.

### Statistical analysis

Categorical variables are expressed as frequencies and percentages, while continuous variables are expressed as means with standard deviations. Univariable logistic regression analysis was conducted to identify factors associated with prolonged hospital stay and in-hospital mortality. Covariates with a p-value of <0.2 in the univariable analysis, age, and sex, which were selected a priori, were included in the multivariable logistic regression model to determine factors independently associated with prolonged hospital stay and in-hospital mortality. For the regression models, missing data were assumed missing at random. Multiple imputation by chained equations was performed to create 20 complete datasets (13). The imputation models included all covariates in the estimation model. Risk estimates are reported as odds ratios (OR) with 95% confidence intervals (CI). A two-tailed p-value of < 0.05 was considered statistically significant. All analyses were performed using Stata 18 statistical package (Statacorp, College Station, TX).

## Results

### General characteristics

From March 2021 to March 2024, 477 consecutive patients were recruited, and 254 (53.2%) were females. The mean age was 60.3 ± 16.5 years. More than half of the participants (55.6%) had hypertension, 20.8% had atrial fibrillation, 17.6% had diabetes, and 14.1% had chronic kidney disease (Table 1). The mean systolic and diastolic blood pressures were 141.2 ± 36.1 mmHg and 93.4 ± 23.4 mmHg, respectively. The mean hemoglobin was 11.5 ± 2.4 g/dl. The mean estimated glomerular filtration rate was 58.4 ± 33.6. All patients performed echocardiography, and the mean left ventricular ejection fraction was 38.2 ± 17.7%.

**Table 1:** General characteristics of the study population.


VARIABLE	FREQUENCY (%) OR MEAN ± SD

Age	60.3 ± 16.5

Sex, Female	254 (53.3)

Atrial fibrillation	99 (20.8)

Previous stroke	21 (4.4)

Hypertension	265 (55.6)

Diabetes mellitus	84 (17.6)

Smoking	

Never	387 (81.1)

Former	54 (11.3)

Current	36 (7.6)

Alcohol	68 (14.3)

Chronic kidney disease	67 (14.1)

COPD	42 (8.1)

HIV	30 (6.3)

SBP	141.2 ± 36.1

DBP	93.4 ± 23.4

Heart rate	102.8 ± 21.3

Hemoglobin (g/dl)	11.5 ± 2.4

eGFR(L/min)	58.4 ± 33.6

Sodium (mmol/l)	140.5 ± 7.2

Potassium (mmol/l)	4.3 ± 2.4

LVEF (%)	38.2 ± 17.7


COPD: chronic obstructive pulmonary disease; DBP: diastolic blood pressure; eGFR: estimated glomerular filtration rate; HIV: human immunodeficiency virus; LVEF: left ventricular ejection fraction; SBP: systolic blood pressure; SD: standard deviation.

### Etiologies of heart failure

The etiologies of heart failure are shown in Table 2. Hypertensive heart disease (41.7%), ischemic heart disease (15%), cor pulmonale (11.9%), and dilated cardiomyopathy (9%) were the most frequent causes of heart failure. Rheumatic heart disease accounted for 4.8% of causes of HF.

**Table 2:** Etiology of heart failure.


VARIABLE	FREQUENCY (%)

Hypertensive heart disease	199 (41.7)

Dilated cardiomyopathy	57 (11)

Ischemic heart disease	72 (15)

Rheumatic heart disease	23 (4.8)

Degenerative valvular disease	26 (5.5)

Cor pulmonale	57 (11.9)

Tachyarrhythmias	20 (4.2)

Infective endocarditis	4 (0.8)

Peripartum cardiomyopathy	6 (1.3)

Pericardial effusion	9 (1.9)

Others	4 (0.8)


### Outcome

The median length of hospital stay was seven days. About half (49.1%) of the patients had a prolonged length of hospital stay. A factor that increased the odds of prolonged length of stay (Table 3) was atrial fibrillation (OR = 2.03, CI: 1.2–3.3; p = 0.005). Factors that reduced the odds of prolonged length of stay were valvular heart disease (OR = 0.48, CI: 0.25–0.90; p = 0.023), systolic blood pressure (OR = 0.99 per 1 mmHg increment, CI: 0.98–0.99; p = 0.005), and plasma sodium (OR = 0.95 per 1 unit increment, CI: 0.92–0.98; p = 0.010). During admission, 57 patients died, giving an in-hospital case fatality of 11.9%. In multivariable logistic regression analysis, factors that reduced the odds of mortality were plasma sodium (OR = 0.92 per 1 unit increment, CI: 0.88–0.96; p < 0.001), systolic blood pressure (OR = 0.98 per 1 mmHg increment, CI = 0.97–0.99; p = 0.033), and glomerular filtration rate (OR = 0.98 per 1 unit increment, CI: 0.96–0.99; p = 0.001). Factors that increased the odds of mortality were treatment with dobutamine (OR = 4.37, CI: 1.11–17.16; p = 0.034) and valvular heart disease (OR = 2.39, CI: 1.01–5.63 p = 0.045) (Table 4).

**Table 3:** Factors associated with prolonged length of stay.


VARIABLE	UNIVARIABLE ANALYSIS		MULTIVARIABLE ANALYSIS	
	
	OR (95%CI)	P VALUE	OR (95%CI)	P VALUE

Age	1.00 (0.99–1.01)	0.563	1.00 (0.99–1.01)	0.941

Male sex	1.02 (0.71–1.46)	0.912	1.16 (0.79–1.71)	0.459

Diabetes Mellitus	1.25 (0.78–2.00)	0.362		

Atrial fibrillation	2.12 (1.34–3.35)	0.001	2.04 (1.24–3.35)	0.005

COPD	1.77 (0.93–3.40)	0.084	1.63 (0.81–3.30)	0.175

SBP	0.99 (0.99–1.00)	0.002	0.99 (0.99–1.00)	0.005

eGFR	1.00 (0.99–1.00)	0.583		

Hemoglobin	0.94 (0.86–1.01)	0.097	0.94 (0.86–1.02)	0.148

Sodium	0.96 (0.93–0.99)	0.004	0.96 (0.93–0.99)	0.010

VHD	0.56 (0.31–0.99)	0.048	0.49 (0.26–0.91)	0.023


COPD: chronic obstructive pulmonary disease; eGFR: estimated glomerular filtration rate; SBP: systolic blood pressure; VHD: valvular heart disease. For continuous variable, odds ratios are reported per 1 unit increment.

**Table 4:** Factors associated with in-hospital mortality.


VARIABLE	UNIVARIABLE ANALYSIS		MULTIVARIABLE ANALYSIS	
	
	OR (95%CI)	P VALUE	OR (95%CI)	P VALUE

Age	1.00 (0.99–1.02)	0.586	1.01 (0.99–1.03)	0.260

Male sex	0.44 (0.24–0.80)	0.007	0.55 (0.28–1.06)	0.074

Diabetes Mellitus	0.74 (0.34–1.62)	0.452		

Atrial fibrillation	1.28 (0.67–2.45)	0.451		

COPD	1.85 (0.81–4.23)	0.143	2.09 (0.81–5.41)	0.130

SBP	0.99 (0.98–0.99)	0.001	0.99 (0.98–0.99)	0.033

eGFR	0.98 (0.97–0.99)	0.001	0.98 (0.97–0.99)	0.001

Hemoglobin	0.93 (0.83–1.05)	0.267		

Sodium	0.92 (0.88–0.96)	< 0.001	0.93 (0.89–0.97)	< 0.001

LVEF	1.00 (0.99–1.02)	0.460		

VHD	2.04 (0.99–4.23)	0.054	2.40 (1.02–5.64)	0.045

Dobutamine	9.63 (3.35–27.71)	< 0.001	4.37 (1.11–17.16)	0.034


COPD: chronic obstructive pulmonary disease; eGFR: estimated glomerular filtration rate; LVEF: left ventricular ejection fraction; SBP: systolic blood pressure; VHD: valvular heart disease. For continuous variable, odds ratios are reported per 1 unit increment.

## Discussion

We sought to determine the contemporary characteristics and the in-hospital outcome of patients hospitalized for decompensated heart failure. The mean age of patients with HF was 60 years, and there was a female predominance. Major comorbid conditions were hypertension, atrial fibrillation, and diabetes mellitus. The most common causes of heart failure were hypertensive heart disease and ischemic heart disease. In-hospital mortality was 11.9%.

More than half (53.2%) of the patients with HF in this cohort were females. This proportion falls in the range reported by other hospital-based studies in SSA HF cohorts, where the proportion of women among heart failure patients ranged between 51–55.5% (14,15). The mean age in this present study was 60 years. This finding is similar to that reported by previous recent studies in Cameroon, where the mean age range was 60–64 years (15,16). It is, however, different from that of patients in the THESUS-HF study published more than a decade ago, where the mean age was 52.3 years (4). Also, a recent study in Ethiopia reported a much younger median age of 34 years, which is explained by the fact that close to half of the patients had rheumatic heart disease (48%), which mostly affects children and young adults in SSA. Because of the high prevalence of RHD in Ethiopia, this center is a referral center that receives patients for interventional treatment for RHD (17). In contrast to SSA, in a large-scale Japanese registry of acute decompensated HF, the mean age of patients was 78 years (18). In other registries in high-income countries, the mean ages were 72.4 years (19) and 73 years (20). This mean age in our study indicates that even though heart failure still affects young patients in low- and middle-income countries compared to high-income countries, the mean age has increased by almost a decade when compared to the mean age in the THESUS-HF (4). This difference is an indication of population aging with increasing life expectancy in SSA.

In this study, a high prevalence of co-morbidities was observed in patients with decompensated HF. The most common comorbid conditions were hypertension (55.6%), atrial fibrillation, (20.8%) and diabetes mellitus (17.6%). About 14% had chronic kidney disease. In an urban center in Cameroon, Anastase et al. reported a high prevalence of hypertension (65%) in the Douala HF registry (16). Other common comorbidities in the Douala HF registry were diabetes (12.4%) and chronic kidney disease (10.7%). The prevalence of atrial fibrillation in our study was similar to that reported in the THESUS-HF (18.2%) and the INTER-HF (17.3%) (4,21). The presence of multiple comorbidities is associated with a higher risk of in-hospital mortality in HF patients (22).

The most common cause of HF in this cohort was hypertensive heart disease (41.7%), with ischemic heart disease (15%) being the second most common etiology of HF. This was similar to findings from the INTER-HF study, which showed that hypertensive heart disease (35%) was the most common cause of HF, followed by ischemic heart disease (20%) (21). Most SSA HF cohorts have shown that hypertensive heart disease is the most common cause of heart failure (23). The THESUS–HF study indicated that HHD and dilated CMP were the leading causes of AHF (4). In the THESUS-HF, ischemic heart disease was not a common cause, representing only 7.7% of the etiologies of HF. The proportion of IHD in our study is twice that reported in the THESUS-HF more than a decade ago (4). In the Douala-HF registry, the proportion of ischemic heart disease was only 4.9% (16). Ischemic heart disease, which is the most common cause of HF in high-income countries, was previously considered rare in SSA. But emerging evidence suggests that the prevalence of ischemic heart disease in SSA is on the rise because of the rapid epidemiological transition with an increasing prevalence of cardiovascular disease risk factors such as hypertension, diabetes, and obesity (5,6). A recent study from Kenya that specifically looked for ischemic heart disease indicated that it was the second most common cause of HF (8). Another study from Kenya reported that ischemic heart disease accounted for more than half (52%) of the etiology in patients with heart failure with reduced ejection fraction. Rheumatic disease represented only 4.8% of the causes of heart failure in our study as the 6^th^ most common etiology. This is lower than the 14.3% reported in the THESUS-HF (4). Previous studies have highlighted a declining contribution of RHD among patients with HF in Africa. The INTER-HF study from five African countries reported an RHD prevalence of 7.7%, which is half the prevalence reported in the THESUS more than a decade ago (21). In contrast, RHD was the most common cause of HF in Ethiopia in two recent studies (30–48.5%). This high prevalence of RHD was explained by the fact that these centers were referral centers where patients received interventional treatment for RHD, thus affecting the distribution of etiologies of heart failure (17,24).

The median length of hospital stay in this study was seven days, and the in-hospital case fatality rate was 11.9%. The median length of stay and in-hospital case fatality were comparable with another study from Cameroon with a length of stay of seven days and in-hospital mortality of 12.8% (15). Other studies, however, reported longer hospital stays of 12 days (25). The in-hospital mortality rate in this study was higher than the 4.2% reported in the THESUS-HF (4). A higher in-hospital mortality rate ranging from 12 to 22.9% was also reported in other cohorts in Africa (17,25,26,27). These differences in mortality and length of stay may be due to disparities in patient characteristics, healthcare infrastructure, or quality of care. The predictors of longer length of hospital stay in our study were atrial fibrillation, low systolic blood pressure, hyponatremia, and valvular heart disease. The predictors of in-hospital mortality in our study were low systolic blood pressure, treatment with dobutamine, low estimated glomerular filtration rate, hyponatremia and valvular heart disease. The INTER-CHF and THESUS-HF reported that kidney dysfunction were predictors of mortality in HF patients (4,21). Hyponatremia has been reported as an independent predictor of prolonged length of hospital stay and in-hospital mortality in HF patients, and hyponatremia was also associated with a lower systolic blood pressure (28). The risk of hyponatremia in patients with HF is associated with the severity of the HF (29). Other studies have reported that inotropic support was associated with a high risk of in-hospital mortality and prolonged length of stay (30). Low admission systolic pressure has also been reported as an independent risk factor of mortality in hospitalized HF patients (31). HF patients with low systolic blood pressure are likely to require inotropic support. In our study, a valvular heart disease etiology was associated with a higher risk of in-hospital mortality and prolonged length of stay. This was in contrast to another study where VHD was associated with a statistically significant longer length of stay, but not in in-hospital mortality (32). This difference can be due to the fact that no patient in our study received any invasive treatment for VHD (surgical or transcatheter). Strategies to improve HF outcomes in Africa may include primary prevention by tackling modifiable risk factors like hypertension and diabetes, improving early diagnosis via accessible screening programs, improving access to evidence-based medications, establishing strong healthcare infrastructures with specialized HF clinics, and performing research focused on the African population to better capture the epidemiology of the disease and treatment requirements in this specific population.

There are some limitations to this study. Several known prognostic factors were not assessed, including biomarkers such as N-terminal Pro-Brain Natriuretic Peptide (NT-proBNP), nutritional status, or pre-admission medications. Furthermore, this was a single-center study conducted in the referral hospital of the region, limiting the generalizability of our findings. Despite these limitations, this study is the largest cohort on HF from a single center in Cameroon. It was a prospective study, which increased the accuracy of the data collected.

## Conclusion

Heart failure patients were older and had a high co-morbidity burden. The cause of HF is still predominantly hypertensive, but there is an increasing contribution of ischemic heart disease. In-hospital mortality was 11.9%, and about half had prolonged length of stay. Predictors of prolonged length of stay were atrial fibrillation, hyponatremia, valvular heart disease, and low SBP. Predictors of in-hospital mortality were low systolic blood pressure, treatment with dobutamine, hyponatremia, decreased glomerular filtration rate and valvular heart disease. There is a need to implement community-based prevention initiatives focusing on hypertension control, lifestyle changes, and prevention and early detection of rheumatic heart disease; to increase access to echocardiography and other diagnostic tools necessary for accurate diagnosis of HF; to ensure availability of guideline-directed therapies; and to establish nurse-driven clinics as effective models for delivering HF care in rural settings where specialist availability is limited.
